# Department of Therapeutics

**Published:** 1894-11

**Authors:** David Cerna

**Affiliations:** Demonstrator of Physiology and Lecturer on the History of Medicine in the Medical Department of the University of Texas, Etc.; Galveston


### Current Medical Literature.

### DEPARTMENT OF THERAPEUTICS.

UNDER THE CHARGE OF DAVID CERNA, M. D., PH. D.,
Demonstrator of Physiology and Lecturer on the History of Medicine in the Medical Department of the University of Texas, etc.

Trichloracetic Acid in Obstinate Epistaxis.—Kossotino (*Vratch*, No. 82, *1894-American Medico-Surgical Bulletin*, October 15, 1894), recommends for the treatment of obstinate epistaxis a 3 per cent. solution of trichloracetic acid, instead of the customary iron chloride. The end of a probe is wrapped with cotton, and this tampon impregnated with the acid solution; and with this all the interior surface of the septum is painted. A 20 per cent. cocaine solution may be added, to overcome the intense burning. The hemorrhage is said to cease immediately; and the advantages of this treatment over the traditional treatment with ferric chloride, is stated to consist in the antiseptic and antiputrescent effect of the acid.

---

EXALGIN IN CHOREA.—In a recent number of the *Archiv für Kinderheilkunde*, Dr. Mettenheimer, of Schwerin, relates two cases of chorea in which he thinks the duration was much shortened by the use of exalgin in doses of from a grain and a half to two grains and a quarter three times a day. The additional treatment employed was that customary in the hospital, and consisted of nutritious diet, malt baths twice a week, iodide of iron and cod-liver oil, and systematic gymnastics.—*New York Medical Journal*, October 20, 1894.

---

COLD BATHING DURING MENSTRUATION.—Depasse (*Gazette de Gynecologie*, October 1, 1894,) believes that cold bathing during menstruation is a beneficial measure, provided women accustom themselves to the treatment by bathing every day for at least eight days before the arrival of the period, when they can continue during the menstrual flow, without any danger. The author relates the case of a very anæmic girl in whom this treatment gave the most satisfactory results. He then refers to the address of Houzel on the same subject, delivered before the recent Boulogne Congress, and in which the following conclusions were set forth: 1. Cold salt water baths facilitate the menstrual flow. 2. They increase the duration of genital life. 3. They increase fecundity in a remarkable manner.

---

THYROID EXTRACT IN THE TREATMENT OF INFANTILE MYXŒDEMA.—In a recent paper read before the American Pædiatric Society, Osler (*New York Medical Journal*, October 20, 1894), reported the successful treatment of a case of infantile myxœdema in which the administration of the thyroid extract produced the happiest results, first, in the entire loss of the cretinoid aspect of the child, in improvement in the color, and in the general nutrition; secondly, in the rapid development, as he had grown four inches in height in a period of fourteen months, and had been able to walk and run about everywhere. Before this he had been carried around in the nurse's arms. In the third place the mental development had been proportionately striking; for fourteen months before his vocabulary had consisted of "mama" and "papa," and now he talked fairly well and said anything he desired. No one meeting the child for the first time would have any idea that there was anything peculiar about him. Although he was still undersized and undeveloped, and not talking as plainly as an ordinary child of its age, the improvement had been very marked and gratifying. At first the child, who had been three years of age and a typical cretin, had taken an amount of the extract corresponding to about a quarter of a gland in each twenty four hours.

---

GUAIACOL IN TUBERCULOSIS.—In an interesting paper, Roland G. Curtin (*University Medical Magazine*, October, 1894) affirms that the class of cases that seem to be most improved by the use of guaiacol is the one where there is a slow progress, a slight rise of temperature, slow digestion from fermentation, and a poor nutrition. The improvement noticed in these cases may be summed up as follows: Improved digestion and nutrition, and diminished breaking down of pulmonary tissue, and consequently less expectoration. In the author's experience, this drug is beneficial in chronic ulceration of the lungs, no matter whether associated with bacillus or not. From this fact, he is satisfied that the *guaiacol has no specific effect upon the tubercle bacillus.* He concludes, finally, that guaiacol is not irritating to the stomach, nor is it so liable to produce irritation of the kidneys or hematuria as creosote. The guaiacol seems to act beneficially on the digestive tract, improving nutrition, and thereby assisting repair. Among the advantages of guaiacol over creosote are, 1st, it is more easily taken; 2d, the process of manufacture insures purity; 3d, you know the exact quantity of the medicinal substance you are administering.

---

THE THERAPEUTIC USES OF CREOSOTE FIFTY YEARS AGO.—William Bodenhamer (*New York Medical Journal*, October 20, 1894) writes an interesting paper on the above subject. The following is a list of formulæ used by the writer:

In the treatment of tuberculosis of the rectum and colon, this mixture was employed:

℞ Ol. creosoti,
Ol. amygdalæ ...........aa 2 drachms.
Tict. opii ....................1 drachm.
Pulv. acaciæ ................4 drachms.
Aq. camphoræ ..............8 ounces.

An ounce of this mixture was injected into the rectum or colon every night on retiring, and every morning immediately after evacuating the bowels.

For chronic catarrh, or ulceration of the rectum, a tablespoonful of the following combination was injected into the rectum night and morning:

℞ Ol. creosoti,

Bals. copaibæ............aa 1 drachm.

Liq. opii sedativi.............1 drachm.

Pulv. acaciæ ......... ....4 drachms.

Aq. camphoræ ...............8 ounces.

The following prescription was found beneficial in the diarrhœa attending pulmonary tuberculosis:

℞ Ol. creosoti ................. 6 minims.
Sp. ammoniæ aromat .........1 drachm.
Aq. menth. piperitæ ..........4 ounces.

A tablespoonful to be taken three times daily.

The following combination is very good in allaying irritability of the stomach in emesis, as in cholera morbus, etc. A tablespoonful to be taken frequently:

℞ Ol. creosoti .................15 minims.
Syr. simplicis ............... 1 ounce.
Aq. menth. piperitæ.......... 5 ounces.

This prescription is good in obstinate leucorrhœa. Inject two tablespoonfuls *per vaginam* twice daily:

℞ Ol. creosoti,
Liq. potassæ ............aa 1 drachm.
Aq. camphoræ .......... .8 ounces.

As an ointment, the following is excellent in burns, fissures, abrasions, chilblains, etc.:

℞ Ol. creosoti ..................1 drachm.
Bismuthi subnit.,
Glycerini .................aa 2 drachms.
Ung. aquæ rosæ .............1 ounce.

The following lotion is good in pruritus ani et vulvæ:

℞ Ol. creosoti ................ 2 drachms.

Acid. oxalici ...... ........½ drachm.

Aq. camphoræ ........... .. 2 ounces.

The writer concludes his paper by hoping that creosote, which is at present placed in the first rank as an excellent and highly prized remedy in tuberculosis, will continue to maintain its high position and stand the test of time, never to be relegated, like so many valuable remedies heretofore have been, to that *limbus* from whose bourn so few ever return.

